# Business of the Quarterly Court

**Published:** 1916-12-09

**Authors:** 


### THE LONDON HOSPITAL.

### Business of the Quarterly Court.

Since the last Quarterly Court, owing to further difficulties experienced in the routine work, the house committee have decided to open only one end instead of two of the surgical and medical out-patients' department every day, and to cut the aural and ophthalmic departments to half their normal number of sessions—that is to say, to two days instead of four. In order to do this, it has been found necessary to strengthen the staff of the receiving-room. Mr. W. H. Forshaw has been appointed chief casualty officer, and a room has been fitted up in the receiving-room where minor operations are performed under gas.

In view of the depletion of the staff it has been arranged that in- or out-patients from the country shall, as far as possible, be referred to their own nearest hospitals; but if resident in the East End they will be treated as usual if urgent.

### Help Given by Medical Officer of Health.

Dr. D. L. Thomas, the Medical Officer of Health for the Borough of Stepney, has come to the assistance of the hospital in a most practical and kind manner, having volunteered to sleep in, so that from 6 o'clock in the evening to 8 in the morning he is on call, to relieve the housemen by dealing with some of the cases brought in during the night.

### Treatment of Venereal Diseases.

Since the last court there have been meetings between the medical authorities of the London County Council and the hospital for the purpose of formulating a workable scheme for the treatment of venereal diseases. Owing to the generosity of the Grocers' Company, the London was the first general hospital in the country enabled to make a serious effort to deal with this scourge, and a much larger scheme than was originally in view has been practically decided on. In consideration of the payment of £3,000 for the first year—January 1, 1917, to December 31, 1917—the hospital will undertake to provide treatment for all cases of venereal diseases coming from the areas of the "participating authorities"—*i.e.*, Essex, Middlesex, Surrey, Hertford, Berks, and London. A department will be opened for the treatment of genito-urinary diseases, the hospital providing four sessions per week, two for men and two for women, the men's sessions remaining open until 7 p.m. Two clinical assistants will be appointed, one senior and one junior; and, if necessary, there will be an increase of the latter by the appointment of local general practitioners. It has been found at the London Hospital that the French and English substitutes of the German drug salvarsan are just as successful for treatment as the original product.

### Wounded Officers.

One hundred wounded officers are at present in the hospital, and it is not improbable that the War Office will request a still further increase of the number of military beds. Although at the moment very few sailors are in, the London is still under contract with the Admiralty to give them the use of 250 beds should the need arise.

Through the good offices of Colonel Woodwark, the War Office official in charge of officers' hospitals, a full-sized billiard-table and all fittings has been presented by Mrs. Mitchison. The table has afforded much pleasure to the wounded officers.

### Bust of Edith Cavell.

Sir George Frampton, the famous sculptor, has most generously presented to the hospital a plaster cast of the bust exhibited last summer at the Royal Academy of the late Nurse Edith Cavell, which will be placed in the sitting-room of the nurses' home under construction in memory of Miss Cavell.

### Shortage of Resident Medical Staff.

After January 1 all the house physicians and house surgeons will have been called up; and as the hospital has almost come to the end of the fourth and fifth year students—men who were permitted by the War Office to complete their studies and become qualified doctors—further changes in carrying on the work will be necessary.

### Nurses' Salaries Increased.

The following increases in nurses' salaries have been decided upon:—

Probationers, whose first year's salary was formerly £12, increased to £17. Second year from £20 to £24.

Staff nurses were formerly paid £24, £25, or £27 per annum. Their salaries now begin at £30 without further increase.

Private staff nurses—first year's salary was £30, rising to £45 by rises of £5 a year. The increase for them will mean that they start at £35, and rise by £5 to £50.

The ward sisters formerly started at £30, and rose by £5 a year to £40. They will now start at £40, and rise to £50 by £5 a year.

No alteration is to be made in the rule which governs the pension scheme of the nursing staff. This rule is that when a nurse reaches forty-five years of age, and has done eighteen years' service at the hospital, she will be entitled to full pay for life.

The total increased expenditure will amount to £3,613 a year; but it is to be hoped that this step in the direction of bettering the position of the hospital nurse will meet with the approval not only of the nursing profession, but of the general public.

### New Member of the House Committee.

Subject to the approval of the court, the house committee have elected Mr. George Whiffen, head of the firm of Messrs. Whiffen and Sons, Limited, manufacturing chemists, to be a member of the house committee and chairman of the dispensary committee.

